# Survival and quality of life in incident systemic sclerosis-related pulmonary arterial hypertension

**DOI:** 10.1186/s13075-017-1341-x

**Published:** 2017-06-02

**Authors:** Kathleen Morrisroe, Wendy Stevens, Molla Huq, David Prior, Jo Sahhar, Gene-Siew Ngian, David Celermajer, Jane Zochling, Susanna Proudman, Mandana Nikpour, the Australian Scleroderma Interest Group (ASIG), Catherine Hill, Sue Lester, Peter Nash, Gian Ngian, Mandana Nikpour, Susanna Proudman, Maureen Rischmueller, Janet Roddy, Joanne Sahhar, Wendy Stevens, Gemma Strickland, Vivek Thakkar, Jenny Walker, Jane Zochling

**Affiliations:** 1 Department of Medicine, The University of Melbourne at St Vincent’s Hospital, 41 Victoria Parade, Fitzroy, 3065 Melbourne, Victoria Australia; 2 Department of Rheumatology St Vincent’s Hospital, 41 Victoria Parade, Fitzroy, 3065 Melbourne, Victoria Australia; 3 Monash University and Monash Health, 246 Clayton Road, Clayton, 3168 Victoria Australia; 4 The University of Sydney at Royal Prince Alfred Hospital, Missenden Road, Camperdown, 2050 NSW Australia; 5 Department of Rheumatology, Menzies Institute for Medical Research, Hobart, Australia; 6 Rheumatology Unit, Royal Adelaide Hospital, North Terrace, Adelaide, SA 5000 Australia; 7 Discipline of Medicine, University of Adelaide, Adelaide, SA 5000 Australia

## Abstract

**Background:**

Pulmonary arterial hypertension (PAH) is a leading cause of mortality in systemic sclerosis (SSc). We sought to determine survival, predictors of mortality, and health-related quality of life (HRQoL) related to PAH in a large SSc cohort with PAH.

**Methods:**

We studied consecutive SSc patients with newly diagnosed (incident) World Health Organization (WHO) Group 1 PAH enrolled in a prospective cohort between 2009 and 2015. Survival methods were used to determine age and sex-adjusted standardised mortality ratio (SMR) and years of life lost (YLL), and to identify predictors of mortality. HRQoL was measured using the Short form 36 (SF-36) instrument.

**Results:**

Among 132 SSc-PAH patients (112 female (85%); mean age 62 ± 11 years), 60 (45.5%) died, with a median (±IQR) survival time from PAH diagnosis of 4.0 (2.2–6.2) years. Median (±IQR) follow up from study enrolment was 3.8 (1.6–5.8) years. The SMR for patients with SSc-PAH was 5.8 (95% CI 4.3–7.8), with YLL of 15.2 years (95% CI 12.3–18.1). Combination PAH therapy had a survival advantage (*p* < 0.001) compared with monotherapy, as did anticoagulation compared with no anticoagulation (*p* < 0.003). Furthermore, combination PAH therapy together with anticoagulation had a survival benefit compared with monotherapy with or without anticoagulation and combination therapy without anticoagulation (hazard ratio 0.28, 95% CI 0.1–0.7). Older age at PAH diagnosis (*p* = 0.03), mild co-existent interstitial lung disease (ILD) (*p* = 0.01), worse WHO functional class (*p* = 0.03) and higher mean pulmonary arterial pressure at PAH diagnosis (*p* = 0.001), and digital ulcers (*p* = 0.01) were independent predictors of mortality.

**Conclusions:**

Despite the significant benefits conferred by advanced PAH therapies suggested in this study, the median survival in SSc PAH remains short at only 4 years.

**Electronic supplementary material:**

The online version of this article (doi:10.1186/s13075-017-1341-x) contains supplementary material, which is available to authorized users.

## Background

Systemic sclerosis (SSc) is a multisystem autoimmune disease, which occurs worldwide with a prevalence ranging from 7/million to 489/million and an incidence ranging from 0.6/million/year to 122/million/year [1]. SSc is characterized by vasculopathy and excessive collagen production, leading to skin and internal organ fibrosis. As there are no effective disease-modifying agents or cure, there is substantial morbidity and mortality in this disease.

Despite an improvement over the last three decades, morbidity and mortality in SSc remain high. This is highlighted in a recent large study showing an age and sex adjusted standardised mortality ratio (SMR) of 4.06 for newly diagnosed SSc patients, with 22.4 and 26.0 years of life lost (YLL) in women and men, respectively [2]. Cardiorespiratory manifestations, in particular pulmonary arterial hypertension (PAH), are the leading cause of SSc-related death [3].

PAH occurs with a prevalence of 8–15% in SSc patients [4, 5]. It is characterised by abnormal vascular proliferation and remodelling, vasoconstriction and thrombosis of the pulmonary vasculature, leading to elevated pulmonary vascular resistance (PVR), ultimately resulting in right heart failure and death [6]. PAH is often asymptomatic in the early phases. Once symptomatic, the average life expectancy without treatment has been 2–3 years [6]. Consequently, annual screening with algorithms incorporating transthoracic echocardiogram (TTE) and pulmonary function tests (PFTs) is recommenced [7].

Historically, treatment options for patients with SSc-PAH are limited [6]. However, in the past decade, with the introduction of new advanced pulmonary vasodilator therapies used as monotherapy or combination therapy, improvement in symptoms, function and survival has been demonstrated [8, 9]. Currently, there are seven PAH-specific therapeutic agents with regulatory approval available for use in Australia. These agents target the prostacyclin pathway (epoprostenol and iloprost), nitric oxide pathway (sildenafil and tadalafil) or the endothelin pathway (ambrisentan, macitentan and bosentan). Although not available for use in Australia, Riociguat is available in other countries. In Australia, the Pharmaceutical Benefit Scheme (PBS) subsidises monotherapy with one of these agents if prescribed by a physician in a government-designated PAH treatment centre. Once on therapy, patients must demonstrate stability or improvement relative to baseline parameters on two tests (6 minute walk distance (6MWD), TTE or repeat right heart catheterization (RHC)). The PAH-specific therapy can be changed if the patient fails to maintain stability on the aforementioned tests. Combined PAH-specific therapy, using two or more drugs with different modes of action can only occur by compassionate access through hospital pharmacies or the manufacturers, or at patients’ own expense. Anticoagulation in the treatment of PAH is a contentious issue in SSc, with some studies showing a survival benefit in patients with idiopathic PAH (iPAH) and connective tissue disease (CTD)-associated PAH [10, 11] and others not showing a survival benefit [12]. Furthermore anticoagulation in SSc is not without risk.

Despite an improvement in survival with these therapies, survival in SSc-PAH remains well below that of iPAH and CTD-PAH [13] with one-year, two-year and three-year survival of 90%, 78% and 56%, respectively compared with one-year, three-year and five-year survival in idiopathic PAH of 92%, 75% and 66%, respectively [13, 14]. Survival in incident SSc-PAH may be below this as these figures are derived from incident and prevalent SSc-PAH cohort data, introducing a survival bias. Not only does SSc-PAH affect patient survival, it also has a significant impact on patients’ functional capacity and health-related quality of life (HRQoL) [15, 16]. We sought to determine survival and HRQoL related to incident SSc-PAH in a large cohort of Australian SSc patients, and to identify predictors of mortality.

## Methods

### Patient cohort

All patients fulfilled either the American College of Rheumatology criteria for SSc or Leroy and Medsger criteria for SSc [17, 18]. Patients included in this analysis were from the Australian Scleroderma Cohort Study (ASCS). The ASCS is a prospective multi-centre study of risk and prognostic factors for cardiopulmonary outcomes in SSc. The ASCS compromises 13 Australian centres and has been approved by the human research ethics committee of each of the participating hospitals (St. Vincent’s Hospital, Melbourne Royal Adelaide Hospital, Monash Medical Centre, Royal Perth Hospital, The Queen Elizabeth Hospital, Sunshine Coast Rheumatology, Prince Charles Hospital, John Hunter Hospital, Royal North Shore Hospital, Royal Prince Alfred Hospital, St George Hospital, Canberra Rheumatology and the University of Tasmania). All patients provide written informed consent at recruitment.

### Inclusion and exclusion criteria

All patients were screened annually for PAH with PFTs and TTE. Any patient identified as at high risk of developing PAH, defined as systolic pulmonary arterial pressure (sPAP_TTE_) of at least 50 mmHg and/or diffusing lung capacity for carbon monoxide (DLCO) <50% predicted with forced vital capacity (FVC) >85% predicted, without adequate explanation on high-resolution computer tomography (HRCT) of the chest or ventilation-perfusion (V/Q) scan of lung or both, underwent RHC.

We included all consecutive adult (age >18 years) SSc patients from the ASCS between June 2009 and June 2015, who were diagnosed with World Health Organization (WHO) Group 1 PAH on RHC (mean pulmonary arterial pressure (mPAP) of at least 25 mmHg and pulmonary arterial wedge pressure (PAWP) <15 mmHg) [19].

Patients were excluded if they had WHO Group 2 or 3 pulmonary hypertension or Group 1 PAH but with co-existing ILD with FVC <60% and abnormal HRCT of the chest. V/Q scanning was used to exclude pulmonary hypertension due to chronic thromboembolism.

### Data collection

Patient demographics, clinical variables and cardiac and pulmonary assessments were obtained from the ASCS database. All physical examination and investigation data were collected within one month of the first RHC, before starting pulmonary vasodilator therapy. Clinical manifestations and autoantibody status were defined as present, if ever present from SSc diagnosis. Scleroderma disease onset and disease duration were defined from the date of onset of the first non-Raynaud manifestation. Autoantibodies measured included anti-nuclear antibodies (ANA), antibodies to extractable nuclear antigens (ENA), anti-RNA polymerase III antibodies, anti-Scl-70 antibody and antiphospholipid antibodies (APLA). TTE was performed according to standardised procedures only at tertiary centres with expertise. Pulmonary involvement was assessed by PFTs and HRCT.

Patient-reported outcome measures were collected annually, including the SSc-specific health assessment questionnaire (SHAQ) and the Medical Outcomes Study Short Form-36 (SF-36), a functional assessment tool and a health-related quality of life measurement tool, respectively, which are both well-validated for use in SSc [20]. These patient-reported outcomes (PROs) were chosen as they are collected annually for each patient in the ASCS.

Demographics and clinical manifestations were compared between SSc patients who developed and those that did not develop PAH. Furthermore, PRO scores were compared between these two groups.

### Outcome variables

The principal outcome variable was all-cause mortality. The date of death was recorded. Where data were available, the exact cause of death was recorded. Patient status (alive or dead) at the time of censoring (January 2016) was confirmed by checking with the treating physician and verified against hospital records. The secondary outcome variables that we evaluated were the most recent SHAQ score and the physical and mental component scores of the SF-36 (PCS and MCS) following PAH treatment.

### PAH therapy and other medications

All specific PAH therapies (endothelin receptor antagonists (ERA), phosphodiesterase-5-inhibitors (PDE5) and prostacyclin analogues) and their combinations (monotherapy or combination therapy) were prescribed at the discretion of the managing physician(s) and these medications were recorded at each visit. Use of other therapeutic agents such as anticoagulation (including indication, date of initiation and target international normalised ratio (INR) for warfarin, date and reason for cessation of anticoagulation), antiplatelet agents, hydroxychloroquine (HCQ), mycophenolate mofetil (MMF), hormone replacement therapy (HRT) and proton pump inhibitors (PPIs) were also at the discretion of the managing physician(s) and were recorded.

### Statistical analysis

Patient characteristics at baseline are presented as mean ± standard deviation for continuous variables and as number (percentage) for categorical variables. All-cause mortality was used for analyses because causes of death could not always be confidently ascribed. Kaplan-Meier (K-M) curves were used to estimate survival in patients with SSc-PAH. One-year, two-year and three-year survival were assessed; date of RHC diagnosis of PAH was considered the baseline from which survival was measured. The log-rank and Wilcoxon tests were used to compare survival curves. The SMR was calculated using the observed deaths in our SSc cohort and the expected deaths in the Australian population, which was sourced from the Australian Bureau of Statistics (ABS). YLL was also calculated based on Australian life expectancy using ABS data.

After testing to ensure proportionality of hazard, Cox proportional hazards regression analyses were used to determine univariable and multivariable predictors of mortality. All variables a with *p* value ≤0.1 in univariable analysis or variables with clinical face validity were selected for inclusion in multivariable analysis. The results were reported as hazard ratios (HR) with accompanying 95% confidence intervals (CI). Mixed effect linear regression was used to identify and quantify determinants of the SHAQ score and the PCS and MCS of the SF-36 following PAH treatment. A two-tailed *p* value ≤0.05 was considered statistically significant. All statistical analyses were performed using STATA 14.0 (StataCorp LP, College Station, TX, USA).

## Results

### Patient characteristics

Of the 1578 SSc patients enrolled in ASCS, 132 patients were diagnosed with incident Group 1 SSc-PAH and included in this study. Patient characteristics by PAH status are summarised in Additional file 1: Table S1. SSc-PAH patient characteristics and haemodynamic measurements are summarised in Table 1. Our SSc-PAH cohort compromised predominantly women (84.9%) with limited disease subtype (limited cutaneous systemic sclerosis (lcSSc)) (68.9%) and a mean (IQR) follow-up duration of 3.8 (1.6–5.8) years since ASCS recruitment. At PAH diagnosis, the mean SSc disease duration was 14.1 ± 11.9 years, with no difference between disease subtypes (*p* = 0.40). Anti-centromere ANA was the most common autoantibody detected (51.6%), followed by APLA (30%). Anti-Scl-70 was infrequent (7.4%).

**Table 1:** Characteristics of patients with SSc-PAH

Characteristic	Mean (± SD), number (percent) or median (IQR)
Total number of patients	132
Female	112 (85%)
Age at PAH diagnosis, years	62.3 (± 10.9)
Disease duration^a^ at PAH diagnosis, years	14.1 (± 11.9)
Status at censoring	
Alive	70 (53.0%)
Dead	60 (45.5%)
Withdrawn	1 (0.8%)
Unable to contact	1 (0.8%)
Race	
Caucasian	112 (84.9%)
Asian	6 (4.6%)
Aboriginal-Islander	1 (0.8%)
Hispanic	1 (0.8%)
Follow-up duration^b^, years (median (IQR))	3.8 (1.6–5.8)
Survival from PAH diagnosis, years (median (IQR))	4.0 (2.2–6.2)
Disease duration^a^ at PAH diagnosis, years	14.4 ± 12.1
Disease subtype	
Limited	91 (68.9%)
Diffuse	30 (22.7%)
MCTD	7 (5.3%)
Autoantibody status	
Anti-centromere pattern ANA	63 (51.6%)
Antiphospholipid antibodies (>ULN)	33 (30%)
RNA polymerase III positive	8 (11.4%)
Scl 70 positive	9 (7.4%)
WHO functional class at time of PAH diagnosis	
Class I	3 (2.3%)
Class II	23 (17.4%)
Class III	79 (59.9%)
Class IV	12 (9.1%)
Baseline 6MWD, m	326.13 (±105.5)
Baseline mRAP, mmHg	8.3 (± 4.3)
Baseline mPAP, mmHg	35.6 (± 10.4)
Baseline PAWP, mmHg	10.5 (± 3.4)
Baseline mCI, L/min/m2	3.2 (± 1.9)
Baseline PVR, Wood units	8.7 (± 3.8)
Presence of a pericardial effusion at PAH diagnosis	24 (18.2%)
Mean DLCO, % predicted mL/min/mmHg	46.6 (± 13.5)
Mean DLCO/VA, % predicted mL/min/mmHg	56.7 (± 20.2)
Medical therapy	
Pulmonary vasodilator therapy^c^	
Monotherapy	91 (68.9%)
Combination therapy	41 (31.1%)
Warfarin therapy^d^	37 (28.5%)
Hydroxychloroquine therapy^d^	12 (9.1%)
Antiplatelet agent^c^	48 (36.9%)
Mycophenolate mofetil therapy^d^	7 (5.4%)
Hormone replacement therapy^d^	16 (12.3%)
Proton pump inhibitor^d^	105 (80.8%)
Home oxygen^d^	28 (21.5%)

*Abbreviations*: *SSc* systemic sclerosis, *PAH* pulmonary arterial hypertension, *MCTD* mixed connective tissue disease, *ANA* antinuclear antibody, *ULN* upper limit of normal, *WHO* World Health Organization, *6MWD* six-minute walk distance, *mRAP* mean right atrial pressure, *mPAP* mean pulmonary arterial pressure, *PAWP* pulmonary artery wedge pressure, *PVR* peripheral vascular resistance, *mCI* mean cardiac index, *DLCO* diffusing capacity of the lung for carbon monoxide, *DLCO/VA* DLCO adjusted for alveolar volume

^a^Disease duration from first non-Raynaud manifestation

^b^Follow-up duration was defined as years from study enrollment

^c^Monotherapy is treatment with a single PAH-specific therapy. Combination therapy is treatment with more than one specific PAH agent from different classes at one time

^d^Treatment ever following the diagnosis of PAH

Despite annual screening, the majority of patients at PAH diagnosis were in WHO functional class II (17.4%) or class III (59.9%) with a mean baseline 6MWD of 326.1 (±105.5) m. Hemodynamics measured at the time of PAH diagnosis showed moderate PAH with an mPAP of 35.6 (± 10.4) mmHg, mean right atrial pressure (mRAP) of 8.3 (± 4.3) mmHg and mean cardiac index (mCI) of 3.2 (± 1.9) L/min/m^2^. Mean DLCO at PAH diagnosis was 46.6% (± 13.5) predicted, and DLCO corrected for alveolar volume (DLCO/VA) was 56.7% (± 20.2) predicted. A pericardial effusion was present at PAH diagnosis in 18.2% of patients.

### Specific PAH therapy

All patients were treated with at least one specific PAH medication. Considering the Australian PBS regulations, in our study, the majority of patients (68.9%) were treated with monotherapy (including sequential therapy) and 31.1% with combination therapy (two or more advanced PAH therapies at the same time). Six patients received upfront combination therapy at the time of PAH diagnosis. The remainder of patients (31 patients (26.5%)) on combination therapy received additional therapy as “add-on” therapy due to functional deterioration. Medications were altered at physician discretion based on failure of the specific PAH therapy or adverse effects.

As monotherapy, bosentan (68.1%) was the most commonly prescribed drug followed by sildenafil (15.9%). Other monotherapy prescribed and its frequency included ambrisentan (8.7%), macitentan (2.9%) and sitaxentan (before its withdrawal) (2%). The most common combination was bosentan and sildenafil (49.1%) followed by bosentan and tadalafil (12.3%). Supplemental home oxygen was used by 21.5% of patients.

Patients treated with combination therapy compared with monotherapy had more severe PAH reflected by a higher mPAP (39.4 (± 11.9) vs. 34.1 (± 10.4) mmHg, *p* = 0.007), mPVR (6.2 (± 3.2) vs. 4.3 (± 2.5) Wood Units, *p* = 0.003), lower DLCO percent than predicted (41.4 (± 11.8) vs. 49.7 (± 13.5), *p* = 0.003) and the presence of a pericardial effusion (36.6% vs. 11.3%, *p* = 0.001) at PAH diagnosis. There was also a trend towards more digital ulcers (68.3% vs. 49.4%, p = 0.06) at PAH diagnosis in those commenced on combination therapy compared with monotherapy. There was no difference in mRAP (*p* = 0.37), mCI (2.7 (± 0.9) vs. 3.5 (± 2.1) L/min/m2, *p* = 0.21), age at PAH diagnosis (*p* = 0.38) or disease subtype (*p* = 0.47) (Additional file 1: Table S2) in combination versus monotherapy.

### Anticoagulation and other medical therapies

In our cohort of SSc-PAH patients, 28.5% were anticoagulated with warfarin, 36.9% were on an antiplatelet agent, 80.8% on a PPI, 12.3% on HRT, 9.1% on HCQ and 5.4% on MMF (for treatment of their skin disease). Nine patients were on both warfarin and aspirin concurrently.

In those who were treated with warfarin, 54.1% were initiated on warfarin specifically for the treatment of PAH and 45.9% were placed on warfarin for another indication following the diagnosis of PAH. Eleven PAH patients had to cease their anticoagulation after their PAH diagnosis due to complications of warfarin therapy including gastrointestinal bleeding (which accounted for 58.3% of reasons for stopping warfarin) and difficulty monitoring the INR (INR target 1.5–2.5).

Patients on anticoagulation had more severe PAH reflected by higher mPVR (6.2 (± 3.6) vs. 4.5 (± 2.5) Wood units, *p* = 0.02), lower mCI (2.4 (± 0.7) vs. 3.7 (± 1.8) L/min/m2, *p* = 0.007), shorter 6MWD (291.3 (± 100.3) vs. 340.2 (± 104.9) m, *p* = 0.01), lower mDLCO (42.3 (± 12.5) vs. 48.6 (± 13.5) mL/min/mmHg, *p* = 0.05) and the presence of a pericardial effusion (36.1% vs. 12.9%, *p* = 0.003) at PAH diagnosis. There was no difference in mRAP (*p* = 0.19), mPAWP (*p* = 0.99), mDLCO/VA (50.0 (± 21.8) vs. 59.4 (±19.3) mL/min/mmHg, *p* = 0.21), mPAP (39.5 (± 14.1) vs. 34.5 (±9.3) mmHg, age at PAH diagnosis (*p* = 0.88), disease subtype (*p* = 0.85) or presence of digital ulcers (*p* = 0.94) (Additional file 1: Table S2) in those who were anticoagulated compared with those who were not.

Of note, 37.5% of patients (6 patients) with a known history of gastric antral vascular ectasia (GAVE), defined as characteristic vascular lesions seen on endoscopy, but without recent bleeding, were anticoagulated with warfarin, while only 27.2% of patients (31 patients) with PAH and no history of GAVE were anticoagulated. This further highlights that many factors, not only GAVE, influence an individual physician’s decision to prescribe anticoagulation in this group of patients.

### Survival in SSc-PAH

**Fig. 1:**
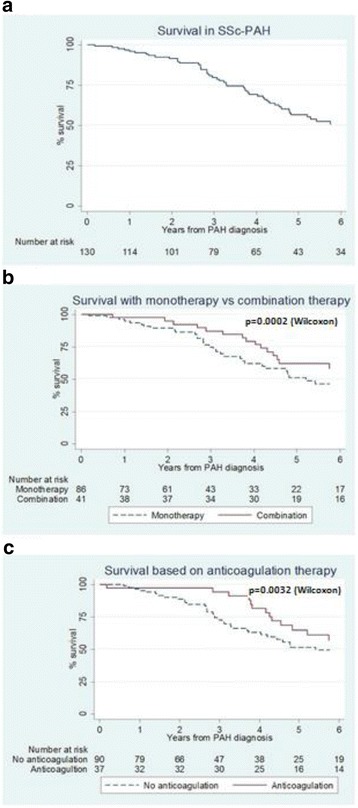
Survival in systemic sclerosis with pulmonary hypertension (*SSc-PAH*). **a** Survival in SSc-PAH. **b** Survival with monotherapy vs combination therapy. **c** Survival based on anticoagulation therapy

SSc-PAH had a significant impact on survival (*p* < 0.001) (Fig. 1). Over a median (± IQR) follow-up of 3.8 (1.6–5.8) years from study enrolment, 60 (45.5%) patients died with a median (± IQR) survival time from PAH diagnosis of 4.0 (2.2–6.2) years. One-year, two-year, three-year and five-year survival was 87.8%, 78.3%, 61.7% and 32.2%, respectively. The age and sex adjusted SMR for patients with SSc-PAH compared with mortality in the general population was 5.8 (95% CI 4.3–7.3). The overall YLL for both male and female patients due to SSc-PAH was 15.2 years (95% CI 12.3–18.1). Men had higher YLLs than women (17.0 years (95% CI 7.7–23.0) compared with 15.4 years (13.8–20.3)). The majority of deaths were directly related to PAH (70%), with PAH being a significant contributor in the remaining causes of death (malignancy (13.3%), gastrointestinal complication (10%), renal (3.3%), and infection (3.3%)).

In univariable analysis (Additional file 1: Table S3), factors associated with mortality included the presence of calcinosis ever, worse WHO functional class, shorter 6MWD, higher mPAP and mPVR and lower DLCO at PAH diagnosis, home oxygen use and lack of PPI use.

Independent predictors of mortality in SSc-PAH in multivariable hazards regression analysis are summarised in Table 2. To ensure model stability, a desired ratio of independent-to-outcome variables was set at one to ten. Older age at PAH diagnosis (HR 1.1, 95% CI 1.0–1.1, *p* = 0.03), presence of mild ILD (HR 2.8, 95% CI 1.4–5.6, *p* = 0.01), worse WHO functional class (HR 2.0, 95% CI 1.1–3.9, *p* = 0.03), higher mPAP at PAH diagnosis (HR 1.1, 95% CI 1.0–1.1 mmHg, *p* = 0.001) and presence of digital ulcers ever (HR 3.1, 95% CI 1.4–7.2, *p* = 0.01) were predictive of mortality. The 6MWD was not predictive of mortality. Using PAH monotherapy as our reference group, the addition of anticoagulation to monotherapy was associated with a trend towards survival benefit (*p* = 0.09). Additionally, PAH combination therapy (all cases were a combination of a PDE5 inhibitor and an ERA) also showed a trend towards a survival benefit compared with monotherapy alone (*p* = 0.10). Furthermore, combination PAH therapy, together with anticoagulation, provided the most significant survival advantage with a 72% reduction in mortality compared with pulmonary vasodilator monotherapy alone (HR 0.28, 95% CI 0.1–0.7, *p* = 0.01).

**Table 2:** Independent predictors of mortality in SSc-PAH determined by multivariable Cox proportional hazard regression analysis

Characteristic	Hazard ratio (95% CI)	*P* value
Age at diagnosis of PAH, years	1.1 (1.0–1.1)	0.03
ILD on HRCT (FVC >60%)	2.8 (1.4–5.6)	0.01
WHO functional class	2.0 (1.1–3.9)	0.03
Pulmonary arterial pressure at PAH diagnosis, mmHg	1.1 (1.0–1.1)	0.001
Digital ulcers present ever	3.1 (1.4–7.2)	0.01
Specific PAH therapies and anticoagulation		
Vasodilator monotherapy only	reference	reference
Vasodilator monotherapy and anticoagulation	0.39 (0.1–1.2)	0.09
Vasodilator combination therapy only	0.49 (0.2–1.2)	0.10
Vasodilator combination therapy and anticoagulation	0.28 (0.1–0.7)	0.01

*Abbreviations*: *SSc* systemic sclerosis, *PAH* pulmonary arterial hypertension, *WHO* world health organization, *ILD* interstitial lung disease, *HRCT* high-resolution computer tomography. *FVC* forced vital capacity, *6MWD* six-minute walk distance, *mRAP* mean right atrial pressure, *mPAP* mean pulmonary arterial pressure, *HCQ* hydroxychloroquine

Kaplan-Meier survival curves (Fig. 1) depict the survival advantage with combination PAH therapy compared with monotherapy (*p* < 0.001) and anticoagulation compared with no anticoagulation (*p* < 0.003). Mean time (± SD) to death was longer for patients who were anticoagulated than those who were not (5.4 (± 2.5) vs. 3.5 (± 2.1), *p* = 0.001) and for those on PAH combination therapy compared to those on monotherapy (5.2 (± 2.8) vs. 3.5 (± 1.9), *p* = 0.02). There was no difference in mean time to death in those with and without APLA on anticoagulation (*p* = 0.68) or those with limited versus diffuse disease subtypes (*p* = 0.56).

### Patient-reported outcome measures in SSc-PAH

In relation to physical function, patients with SSc-PAH had significantly lower SHAQ scores indicating significant functional limitation in their daily activities compared with SSc patients without PAH (Table 3). Determinants of a better SHAQ score using mixed effects linear regression included older age at PAH diagnosis (coefficient −0.1, 95% CI 0.1 to 0.1, *p* = 0.02) while the presence of GIT manifestations (coefficient 0.7, 95% CI −2.7 to −0.1, *p* = 0.04) was associated with a worse SHAQ score over time. Neither PAH-specific therapy nor anticoagulation was associated with a significant change in the total SHAQ score after treatment (Table 4).

**Table 3:** Patient-reported outcomes in patients with SSc-PAH compared to patients with SSc without PAH

Outcomes	PAH	No PAH	*P* value
SHAQ domain^a^			
Patient number	132	1447	
Total score	3.2 ± 1.7	3.3 ± 2.1	0.74
Breathing	4.9 ± 2.5	2.3 ± 2.4	<0.001
Digital ulcers	1.9 ± 2.5	1.4 ± 2.4	0.02
Intestinal	2.8 ± 2.5	2.2 ± 2.4	0.002
Patient global assessment	5.1 ± 2.2	3.6 ± 2.4	<0.001
Pain	3.9 ± 2.6	3.4 ± 2.6	0.03
Vascular (RP)	3.3 ± 2.5	2.7 ± 2.5	0.01
SF-36 domain^b^			
Physical functioning	35.7 ± 23.8	57.5 ± 28.9	<0.001
Role limitation, physical	27.2 ± 39.1	49.2 ± 43.4	<0.001
Role limitation, emotional	55.9 ± 44.9	67.3 ± 40.4	0.05
Social functioning	64.2 ± 27.7	70.5 ± 26.9	0.07
Mental health	66.1 ± 21.1	68.9 ± 20.2	0.28
Energy/vitality	38.6 ± 22.2	47.1 ± 24.1	0.01
Bodily pain	55.3 ± 28.8	60.7 ± 27.9	0.10
General health perception	36.6 ± 20.5	46.2 ± 22.7	0.01
Physical component score	31.7 ± 8.7	38.9 ± 11.6	<0.001
Mental component score	46.3 ± 10.7	46.3 ± 10.4	0.48

Systemic sclerosis (SSc)-specific health assessment questionnaire (SHAQ) and Medical Outcomes Study Short Form-36 (SF-36) values are based on the average of all instrument scores collected annually in the database. For those with pulmonary hypertension (PAH), this includes only those scores that have been collected since PAH diagnosis. For those without PAH this includes all scores within the database for these patients. Raynaud's phenomenon (RP)

^a^The SHAQ is a generic instrument measuring functional outcome validated for use in SSc. The score ranges from 0 to 10, with 0 being no functional limitation and 10 being severe functional limitation

^b^The SF-36 form is a 36-item scale that measures eight domains of health status. The final score is standardised to the general population normative score of 50. The final score for each domain lies between 0 and 100, with 0 being the worst possible health and 100 the best possible health

**Table 4:** Impact of PAH-specific therapy and anticoagulation on health-related quality of life scores in SSc-PAH determined through mixed effects linear regression modeling

Variables	Coefficient (95% CI)	*P* value
Determinants of SF-36 physical component score		
Female gender	0.4 (−3.9, 4.7)	0.85
Age at PAH diagnosis, years	0.1 (−0.1, 0.2)	0.29
Diffuse disease subtype	−0.3 (−3.8, 3.2)	0.87
Combination therapy	1.7 (−1.5, 5.0)	0.29
Anticoagulation therapy	−3.7 (−7.1, −0.3)	0.03
GIT involvement^a^	4.7 (0.9, 8.6)	0.01
Digital ulcers^a^	−3.7 (−7.2, −0.1)	0.04
Determinants of SF-36 mental component score		
Female gender	−3.5 (−8.6, 1.6)	0.18
Age at PAH diagnosis, years	−0.1 (−0.2, 0.1)	0.67
Diffuse disease subtype	−1.1 (−5.2, 3.0)	0.59
Combination therapy	5.2 (1.3, 9.1)	0.01
Anticoagulation therapy	−2.5 (−6.6, 1.6)	0.22
GIT involvement^a^	−1.6 (−6.2, 2.9)	0.48
Digital ulcers^a^	2.8 (−1.5, 7.1)	0.19
Determinants of SHAQ score		
Female gender	0.3 (−0.5, 0.9)	0.46
Age at PAH diagnosis, years	−0.1 (−0.1, −0.1)	0.02
Diffuse disease subtype	−0.5 (−1.1, 0.2)	0.13
Combination therapy	−0.3 (−0.8, 0.3)	0.38
Anticoagulation therapy	0.3 (−0.3, 0.9)	0.28
GIT involvement^a^	0.7 (0.1, 1.3)	0.03
Digital ulcers^a^	−0.1 (−0.7, 0.5)	0.79

*Abbreviations*: *SSc* systemic sclerosis, *PAH* pulmonary arterial hypertension, *GIT* gastrointestinal involvement, *SHAQ* scleroderma health assessment questionnaire

^a^Disease manifestations present if present at PAH diagnosis or at any follow-up visit following PAH diagnosis

SSc-PAH patients had lower HRQoL scores across a number of domains of the SF-36 at PAH diagnosis, particularly in physical functioning, role-physical and general health and vitality, compared with the US normative mean of 50, indicating decreased HRQoL [21] and significantly lower SF-36 scores than SSc patients without PAH (Table 3). Determinants of worse SF-36 PCS using mixed effects linear regression included the presence of digital ulcers (coefficient −3.7, 95% CI −7.2 to −0.1, *p* = 0.04) and warfarin therapy (coefficient −3.7, 95% CI −7.1 to −0.3; *p* = 0.03). The presence of GIT manifestations was associated with better SF-36 PCS score (coefficient 4.7, 95% CI 0.9 to 8.6, *p* = 0.01). Combination therapy was not associated with a significant change in PCS scores after treatment (Table 4). Determinants of improved SF-36 MCS using mixed effects linear regression included treatment with combination PAH therapy (coefficient 5.2, 95% CI 1.3–9.1, *p* = 0.01) (Table 4).

## Discussion

In our SSc-PAH cohort, the median overall survival was only 4 years with mortality of 45.5% over a follow-up period from study enrolment averaging 3.8 years. The one-year, two-year and three-year survival was 87.8%, 78.3% and 61.7%, respectively. Our results are similar to those in a recent French study (90%, 78% and 50% survival) and lower than in a recent American study (93%, 88% and 75%), both of which also prospectively studied survival in a cohort with incident SSc-PAH [13, 22]. The majority of patients in the American study had New York Heart Association (NYHA) functional Class II disease at PAH diagnosis, which may account for the higher three-year survival in that study. Additionally, combination PAH therapy is more readily available in America than in Australia, which may partly explain the better survival in America. The NYHA functional class at PAH diagnosis was similar to ours in the French study. Mortality rates in the literature vary depending on whether cohorts include patients with incident disease only or a combination of those with incident and prevalent disease, with the potential of underreporting mortality in cohorts with prevalent disease due to survival bias. To our knowledge, this is the first paper to quantify YLL associated with SSc-PAH.

Of concern in our cohort, was that despite annual screening for PAH, the majority of patients were in WHO functional Class III at PAH diagnosis. This may be because our screening algorithm missed patients with early or mild PAH without a markedly elevated RVSP, which may help to explain the relatively advanced stages of PAH observed in our study. It is becoming increasingly recognised that WHO functional class is an independent predictor of mortality [13, 23], as was shown in our cohort. The 6MWD was not associated with mortality in our study despite previous Australian data showing an association [11], suggesting that 6MWD is a non-specific outcome measure for PAH, and affected by the other complications of SSc.

Another independent predictor of mortality in our cohort included older age at PAH diagnosis, which has been reported in the literature to be a predictor of poor survival, with one study indicating that patients diagnosed with PAH over the age of 60 years had threefold higher mortality risk than those diagnosed under 60 years of age [22].

Certain clinical manifestations such as the presence of digital ulcers, calcinosis and telangiectasia have been reported to predict those patients at a higher risk of developing PAH [24–26]. In our study, the presence of digital ulcers was associated with greater mortality in SSc-PAH, which may represent a common underlying pathogenic mechanism involving endothelial dysfunction. Alternatively, it may be an indicator of recurrent infections or perhaps it identifies patients with a more severe vascular phenotype with obliterative vasculopathy involving the macrovasculature and microvasculature, manifesting in PAH, digital ischaemia, ulcers and amputation.

The presence of moderate or severe ILD is in itself a risk factor for death in SSc [27, 28]. In our cohort of patients, we excluded those with severe ILD defined as FVC <60% and HRCT showing ILD, in whom PAH may have occurred secondary to ILD. However, we included patients with Group 1 PAH and co-existent mild ILD defined by FVC > 60% and mild abnormalities or no abnormalities on HRCT. Mild ILD was present in 51 patients (38.6%) in our cohort and was predictive of death in SSc-PAH. We postulate that the co-existence of these two clinical manifestations could be due to shared underlying pathogenic mechanisms leading to a more severe clinical phenotype or that the occurrence in the lung of two independent pathologic conditions increases the risk of death.

There is evolving evidence to suggest that compared with monotherapy, the treatment of PAH with combination therapy is associated with improved survival in PAH. In small randomised trials and observational studies, combination therapy by means of “add-on” PAH therapy has consistently shown a survival benefit in PAH [11, 29, 30]. More recently, the treatment of PAH with upfront combination therapy compared with monotherapy showed not only a survival benefit, but also reduced hospitalisation for worsening PAH and disease progression [9].

Anticoagulation in PAH remains controversial despite some observational studies showing a survival benefit [1, 10]. The survival benefit is particularly apparent in patients with iPAH as shown in the COMPERA study [31], Interestingly, this study did not show a survival benefit with anticoagulation in the non-idiopathic PAH group, which included patients with PAH secondary to CTD, congenital heart disease and portopulmonary hypertension. This may be due to the inclusion of these subgroups all within one category [31]. Furthermore, the REVEAL study showed no significant survival advantage in iPAH or SSc-PAH with the addition of anticoagulation [12]. Therefore, we believe that further research is required to assess the role of anticoagulation in PAH specifically associated with SSc.

Australian data collected between 2002 and 2009 identified a survival advantage with warfarin therapy in patients with CTD-PAH, the majority of whom had SSc [11]. In our study survival was similar if not worse than in patients diagnosed and treated between 2002 and 2009. Two reasons may explain this. First, our study included only patients with incident PAH whereas the previous study included both prevalent and incident cases, thus increasing potential survival bias. Second, survival in SSc-PAH is worse than that in CTD-PAH due to other autoimmune rheumatic diseases such as systemic lupus erythematosus and rheumatoid arthritis, so the overall survival may have been increased due to the inclusion of CTD-PAH “survivors”.

There was improved survival in both studies with anticoagulation as an adjunct to PAH therapy compared with PAH therapy in isolation, despite patients on anticoagulation having had more severe PAH at baseline. We propose that the benefit of anticoagulation relates to the prevention of further micro-thrombotic phenomena occurring in the pulmonary vasculature, which likely plays an important role in the underlying pathogenesis of SSc-PAH. The results of our study provide a rationale for a randomised controlled trial evaluating anticoagulation as adjunct therapy in SSc-PAH, which the trial Systemic Sclerosis Pulmonary Hypertension Intervention with Apixaban (SPHInX) (ACTRN12614000418673) aims to resolve.

MMF has been shown to be associated with improved survival in small groups of patients with SSc-PAH [32]. We were not able to replicate these findings as we only had seven patients on MMF in our cohort, which may be explained by the limited availability and expense of MMF in Australia outside of tertiary hospitals until 2015. We were also interested in the relationship between the use of HCQ and survival in SSc-PAH given its anti-platelet effect. Our study identified a trend towards improved survival with HCQ, but this was not statistically significant. With only 12 patients on HCQ in our cohort, our study may not have been sufficiently powered to show such an association.

Not only does SSc-PAH affect survival, it also has a significant impact on patients’ functional capacity and HRQoL [15, 16]. Functional limitations, as captured by the SHAQ, were maintained over time with PAH therapy in our cohort, without a significant improvement in any specific domain, which is consistent with the literature [33, 34].

The physical component of HRQoL, as determined by the SF-36 PCS, was also not improved with combination PAH therapy in our cohort. However, combination PAH therapy was associated with a significant improvement in the mental component of HRQoL as measured using the SF-36 MCS. Reduction in mortality with minimal change in HRQoL has been previously reported in patients with CTD-PAH who are on PAH therapy [30, 34, 35]. The lack of improvement in the physical component of HRQoL following PAH treatment may reflect the complex, multifactorial and individual nature of HRQoL, which is impacted upon by a variety of factors that are difficult to measure and adjust for.

We recognise that there are limitations to our study. Lead-time bias may have contributed to the improvement in survival in patients diagnosed as a result of annual screening, with earlier implementation of PAH-specific therapy and anticoagulation. We excluded patients with co-existent PAH and severe ILD to ensure we captured only those patients with Group 1 PAH. Therefore we did not assess whether severe ILD contributed to mortality, although we assume it would, as mild ILD was a predictor of worse survival. In addition, treatment was not randomised; rather, it was prescribed at the individual physician’s discretion as there is no standard nationwide protocol for the SSc-PAH treatment.

## Conclusion

Despite advanced therapy, the median survival in SSc-PAH is only 4 years. In our study, the addition of anticoagulation to standard combination therapy was associated with a significant survival advantage, further pointing to mechanisms involving endothelial abnormalities and small vessel thrombosis in the pathogenesis of PAH. Although there was no significant improvement in physical function or in the physical components of HRQoL scores over time, these remained stable with PAH therapy.

## Additional file

Additional file 1: Table S1. Patient characteristics by PAH status. **Table S2.** Baseline haemodynamics of SSc-PAH patients according to PAH therapy and anticoagulation. **Table S3.** Predictors of mortality in SSc-PAH determined by univariable analysis. **Figure S1.** Flowchart of SSc patient inclusion in the study. (DOC 85 kb)

## Abbreviations

6MWD: Six-minute walk distance
ABS: Australian Bureau of Statistics
ANA: Anti-nuclear antibodies
APLA: Antiphospholipid antibodies
ASCS: Australian Scleroderma Cohort Study
ASIG: Australian Scleroderma Interest Group
CTD: Connective tissue disease
DLCO: Diffusing lung capacity for carbon monoxide
ENA: Extractable nuclear antigens
ERA: Endothelin receptor antagonists
FVC: Forced vital capacity
GAVE: Gastric antral vascular ectasia
HCQ: Hydroxychloroquine
HRCT: High-resolution computer tomography
HRQoL: Health-related quality of life
HRT: Hormone replacement therapy
ILD: Interstitial lung disease
INR: International normalised ratio
iPAH: Idiopathic pulmonary arterial hypertension
lcSSc: Limited cutaneous systemic sclerosis
Mci: mean cardiac index
MCS: Mental component scores
MMF: Mycophenolate mofetil
mPAP: Mean pulmonary arterial pressure
mRAP: Mean right atrial pressure
NYHA: New York Heart Association
PAH: Pulmonary arterial hypertension
PAWP: Pulmonary arterial wedge pressure
PBS: Pharmaceutical Benefit Scheme
PCS: Physical component scores
PDE5: Phosphodiesterase-5-inhibitors
PFT: Pulmonary function test
PPIs: Proton pump inhibitors
PROs: Patient-reported outcomes
PVR: Pulmonary vascular resistance
RFTs: Respiratory function tests
RHC: Right heart catheterization
RVSP: Right ventricular systolic pressure
SF-36: Medical Outcomes Study Short Form-36
SHAQ: SSc-specific health assessment questionnaire
SMR: Standardised mortality ratio
SSc: Systemic sclerosis/scleroderma
TTE: Transthoracic echocardiogram
V/Q scan: Ventilation-perfusion scan
YLL: Years of life lost
