# Supplementary material for: Survival and quality of life in incident systemic sclerosis-related pulmonary arterial hypertension
Source: Arthritis Res Ther. 2017 Jun 2;19:122. doi: 10.1186/s13075-017-1341-x (PMC5457656; doi:10.1186/s13075-017-1341-x)
Supplement: Additional file 1: Table S1. — Patient characteristics by PAH status. Table S2. Baseline haemodynamics of SSc-PAH patients according to PAH therapy and anticoagulation. Table S3. Predictors of mortality in SSc-PAH determined by univariable analysis. Figure S1. Flowchart of SSc patient inclusion in the study. (DOC 85 kb) [file 13075_2017_1341_MOESM1_ESM.doc]

Supplementary Material

Table S1. Patient characteristics by PAH status

|  | **PAH** | **No PAH** | ***p* value** |
| --- | --- | --- | --- |
|  | **mean ± SD or %** | **mean ± SD or %** |  |
| Number of patients | 132 | 1446 |  |
| Age at recruitment, years | 62.7 ± 10.3 | 56.6 ±12.8 | <0.001 |
| Disease duration at recruitment, years | 14.4 ± 12.0 | 10.8 ± 9.9 | 0.16 |
| Female | 85% | 87% | 0.56 |
| Limited disease subtype | 68.9% | 73.0% | 0.61 |
| Anti-centromere pattern ANA | 51.6% | 44.3% | 0.10 |
| Scl 70 +ve | 7.7% | 14.5% | 0.04 |
| RNA polymerase III +ve | 11.4% | 12.6% | 0.78 |
| Digital ulcers ever | 57.1% | 41.9% | 0.001 |
| Telangiectasia ever | 92.7% | 80.7% | 0.001 |
| Calcinosis ever | 57.9% | 34.1% | <0.001 |
| ILD | 38.6% | 24.1% | 0.007 |
| Current status  Alive  Dead | 53.0%  45.5% | 78.2%  8.7% | <0.001 |
| WHO Functional Class  Class I  Class II  Class III  Class IV | 2.3%  17.4%  59.9%  9.1% | 16.5%  36.5%  42.0%  5.0% | <0.001 |

Table S2. Baseline haemodynamics of SSc-PAH patients according to PAH therapy and anticoagulation

| **Variables** | **Monotherapy** | **Combination therapy** | **p-value** |
| --- | --- | --- | --- |
| 6MWD, m | 329.9±104.7 | 319.4±106.4 | 0.66 |
| Baseline mRAP, mmHg | 8.5±4.2 | 8.1±4.6 | 0.37 |
| Baseline mPAP, mmHg | 34.1±10.4 | 39.4±11.9 | 0.007 |
| Baseline PAWP, mmHg | 10.8±3.4 | 9.7±3.3 | 0.05 |
| Baseline mCI, L/min/m2 | 3.5±2.1 | 2.7±0.9 | 0.21 |
| Baseline PVR, Wood Units | 4.3 ± 2.5 | 6.2 ±3.2 | 0.003 |
| Pericardial effusion | 9 (11.3%) | 15 (36.6%) | 0.001 |
| DLCO, mL/min/mmHg | 49.7±13.5 | 41.4±11.8 | 0.003 |
| DLCO/VA, mL/min/mmHg | 60.1±19.7 | 50.3±19.3 | 0.23 |
| Digital Ulcers ever | 44 (49.4%) | 28 (68.3%) | 0.06 |
| Age at PAH diagnosis | 62.9±10.4 | 60.7±10.8 | 0.38 |
| Disease subtype  Diffuse  Limited | 22 (27.2%)  59 (72.8%) | 8 (25.2%)  30 (78.9%) | 0.47 |
| Time to death | 3.5±1.9 | 5.2±2.8 | 0.02 |
| **Variables** | **Anticoagulation** | **No anticoagulation** | **p-value** |
| 6MWD, m | 291.3±100.3 | 340.2±103.9 | 0.01 |
| Baseline mRAP, mmHg | 9.0±3.8 | 8.1±4.5 | 0.19 |
| Baseline mPAP, mmHg | 39.5±14.1 | 34.3±9.5 | 0.13 |
| Baseline PAWP, mmHg | 10.6±3.3 | 10.5±3.5 | 0.99 |
| Baseline mCI, L/min/m2 | 2.4±0.7 | 3.7±1.8 | 0.007 |
| Baseline PVR, Wood Units | 6.2±3.6 | 4.5±2.5 | 0.02 |
| Pericardial effusion | 13 (36.1%) | 11 (12.9%) | 0.003 |
| DLCO, mL/min/mmHg | 42.3±12.5 | 48.6±13.5 | 0.05 |
| DLCO/VA, mL/min/mmHg | 50.0±21.8 | 59.4±19.3 | 0.21 |
| Time to death | 5.4±2.5 | 3.5±2.1 | 0.001 |
| Digital Ulcers ever | 20 (55.6%) | 51(54.9%) | 0.94 |
| Age at PAH diagnosis | 61.4±11.4 | 62.5±10.2 | 0.88 |
| Disease subtype  Diffuse  Limited | 8 (26.7%)  25 (28.4%) | 22 (73.3%)  63 (71.6%) | 0.85 |

Abbreviations: 6MWD six minute walk distance, mRAP mean right atrial pressure, mPAP mean pulmonary arterial pressure, PAWP pulmonary artery wedge pressure, PVR peripheral vascular resistance, mean cardiac index (mCI), DLCO diffusing capacity of the lung for carbon monoxide, DLCO/VA adjusted diffusing

Table S3. Predictors of mortality in SSc-PAH determined by univariable analysis

| **Characteristic** | **Hazard Ratio (95%CI)** | **p-value** |
| --- | --- | --- |
| Male gender | 1.2 (0.6-2.4) | 0.63 |
| Disease duration* at PAH diagnosis, years | 1.0 (0.9-1.1) | 0.19 |
| Age at PAH diagnosis, years | 1.0 (0.9-1.1) | 0.05 |
| Caucasian race | 1.2 (0.4-3.9) | 0.73 |
| Diffuse disease subtype | 1.3 (0.7-2.4) | 0.45 |
| Autoantibody  RNA polymerase III positive  Scl-70  ACA  APLA | 1.4 (0.4-4.7)  1.2 (0.5-3.2)  0.7 (0.4-1.2)  0.6 (0.3-1.1) | 0.63  0.69  0.15  0.09 |
| Disease characteristics  ILD (FVC > 60%)  Digital ulcers  Telangiectasia  Calcinosis  Pericardial effusion | 1.7 (0.9-2.9)  1.4 (0.8-2.7)  1.1 (0.3-3.5)  0.5 (0.3-0.9)  1.5 (0.9-2.8) | 0.06  0.27  0.89  0.05  0.17 |
| WHO Functional Class at PAH diagnosis | 1.8 (1.1-3.0) | 0.02 |
| Baseline 6MWD, m | 0.9 (0.9-0.9) | 0.001 |
| Haemodynamics  Baseline mRAP, mmHg  Baseline mPAP, mmHg  Baseline PAWP, mmHg  Baseline cardiac index, L/min/m2  Baseline PVR, Wood Units  Baseline DLCO, % | 1.1 (0.9-1.1)  1.0 (1.0-1.1)  0.9 (0.9-1.0)  0.6 (0.3-1.0)  1.1 (1.0-1.2)  0.9 (0.9-1.0) | 0.06  0.01  0.16  0.07  0.03  0.05 |
| Medical Therapies**  Home oxygen  Combination vasodilator therapy  Anticoagulation therapy  HCQ therapy  Antiplatelet agent  Hormone replacement therapy  Proton pump inhibitor therapy | 1.8 (0.9-3.1)  0.7 (0.4-1.3)  0.9 (0.5-1.6)  0.2 (0.1-1.2)  0.7 (0.4-1.3)  0.8 (0.4-1.8)  0.3 (0.2-0.7) | 0.05  0.28  0.65  0.07  0.31  0.59  0.01 |
| Specific PAH therapies and anticoagulation  Vasodilator monotherapy only  Vasodilator monotherapy and anticoagulation  Vasodilator combination therapy only  Vasodilator combination therapy and anticoagulation | Reference  0.7 (0.2-1.8)  0.6 (0.3-1.3)  0.5 (0.3-1.2) | 0.46  0.18  0.13 |

Abbreviations: PAH pulmonary arterial hypertension, ILD interstitial lung disease, 6MWD six minute walk distance, mRAP mean right atrial pressure, mPAP mean pulmonary arterial pressure, PAWP pulmonary artery wedge pressure, PVR peripheral vascular resistance, mean cardiac index (mCI), DLCO diffusing capacity of the lung for carbon monoxide, DLCO/VA adjusted diffusing, HCQ Hydroxychloroquine

*disease duration from first non-Raynaud manifestation ** Treatment ever following the diagnosis of PAH

Figure S1 Flowchart of SSc patient inclusion in the study

| Total SSc cohort  n = 1578 |
| --- |
|  |
| SSc with incident PAH  (n=132) |

| Excluded: n= 1446  Consisting of:   - SSc without PAH: n=1390 - SSc patients with severe ILD: n=39 - SSc patients with Group 2 PH: n=17 |
| --- |
